# “Morphological, Functional, and Molecular Progression of Blood-Brain Barrier Dysfunction in Alzheimer’s Disease”

**DOI:** 10.1007/s12031-026-02610-0

**Published:** 2026-10-14

**Authors:** Karen León-Arcia, Humberto Martínez-Orozco, Magdalena Guerra-Crespo, Sofía Y. Díaz-Miranda, Luis O. Soto-Rojas

**Affiliations:** 1 Departamento de Neurobiología del Desarrollo y Neurofisiología, Instituto de Neurobiología, Universidad Nacional Autónoma de México (UNAM), Querétaro, 76230 México; 2 Laboratorio de Investigación en Neurociencias, Envejecimiento y Neurodegeneración (LINEN), Facultad de Estudios Superiores de Iztacala, UNAM, Tlalnepantla de Baz, 54090 México; 3 PhD Student, Posgrado en Ciencias Biológicas, Unidad de Posgrado, UNAM, Edificio A, 1er Piso, Circuito de Posgrado, Ciudad Universitaria, Ciudad de México, 04510 México; 4 Laboratorio de Medicina Regenerativa, Departamento de Fisiología, Facultad de Medicina, UNAM, Ciudad de México, 04510 México

**Keywords:** Blood-brain barrier, Neurovascular unit, Alzheimer’s disease, Brain insulin resistance, Vascular biomarkers, Pericytes

## Abstract

Blood-brain barrier (BBB) dysfunction is increasingly recognized as an early pathological event in sporadic Alzheimer’s disease (AD), which represents over 95% of cases. Vascular risk factors and cerebral insulin resistance are considered potential contributors to its initiation. This review synthesizes evidence available through March 2026 on the temporal progression of morphological, functional, and molecular BBB alterations in AD patients and experimental models. This review emphasizes the proposed role of BBB disruption in perpetuating neurodegeneration through feedback loops involving pericyte damage, hypoxia, perivascular inflammation, and dysregulation of amyloid-β transport systems. By integrating data from neuroimaging, fluid biomarkers, and post-mortem studies, available evidence suggests that BBB alterations can be detected from preclinical stages, most consistently in the hippocampus, and may exhibit sex-specific differences. The review compares findings from familial transgenic models with the more limited characterization available in sporadic AD models (notably the intracerebroventricular streptozotocin model), and highlights key knowledge gaps regarding temporal dynamics. This synthesis supports further exploration of strategies to preserve BBB integrity. An integrated vascular approach may usefully complement current amyloid- and tau-centered therapies, particularly in sporadic AD.

**Supplementary Information:**

The online version contains supplementary material available at https://doi.org/10.1007/s12031-026-02610-0.

## Introduction

Alzheimer’s disease (AD) is the most prevalent cause of dementia in older adults and represents one of the most significant public health challenges of the 21st century. Two primary forms are distinguished: familial AD, which is genetic in origin and accounts for less than 5% of cases, caused by autosomal dominant mutations in the *amyloid precursor protein (APP)*, *presenilin (PSEN) 1*, or *PSEN2* genes; and sporadic AD, which affects more than 95% of patients. Sporadic AD has a multifactorial etiology in which vascular and metabolic risk factors, such as hypertension, type 2 diabetes, and dyslipidemia, interact with genetic susceptibility. The *apolipoprotein E ε4 allele* (*APOEε4*) is the major genetic risk factor, while additional loci, including *triggering receptor expressed on myeloid cells 2* (*TREM2*), *sortilin-related receptor 1* (*SORL1*), and *clusterin* (*CLU*), also contribute to risk (Armstrong 2019; Frisoni et al. 2022; Andrade-Guerrero et al. 2023; Xiaopeng et al. 2025; Wu et al. 2026). Type 2 diabetes is a well-established risk factor for sporadic AD, and brain insulin resistance may contribute to cerebrovascular dysfunction and blood-brain barrier (BBB) impairment (Rhea and Banks 2019; Kellar and Craft 2020). These vascular and metabolic risk factors are linked to the onset of cerebrovascular dysfunction, which may contribute to both disease onset and progression.

The “two-hit” vascular hypothesis proposes that initial damage to cerebral vessels (first hit) compromises BBB integrity, leading to hypoperfusion and reduced clearance of the amyloid-beta (Aβ) peptide; this facilitates toxic Aβ accumulation (second hit) and subsequent neurodegeneration. This interaction between the vascular and amyloidogenic pathways may operate independently or synergistically, suggesting that BBB dysfunction is not merely a late epiphenomenon but a dynamic process (Nelson et al. 2016; Rius-Pérez et al. 2018; De La Torre 2018). According to the National Institute on Aging and Alzheimer’s Association (NIA-AA) criteria, the disease is defined as a biological continuum spanning three main stages: the preclinical stage, characterized by detectable pathophysiological changes via biomarkers without cognitive impairment; the prodromal stage or mild cognitive impairment (MCI) due to AD; and the dementia phase (Jack et al. 2024). Under this paradigm, vascular alterations appear to be detectable and may be amplified starting from preclinical stages, particularly in sporadic AD (Nelson et al. 2016; Rius-Pérez et al. 2018; De La Torre 2018).

However, the available literature remains fragmented. Most reviews focus on isolated findings, either in humans or in transgenic models that primarily mimic the familial form, without critically integrating the chronology of morphological, functional, and molecular BBB changes or systematically comparing them across the different forms of the disease (Sweeney et al. 2018; Kurz et al. 2022; Nehra et al. 2022; Chen et al. 2023b). Recent advances, such as reported sexual dimorphism in vascular response (Moon et al. 2021; Li et al. 2024; Lin et al. 2024), the potential contribution of cerebral insulin resistance (Rhea and Banks 2019; Padovani et al. 2025), and the identification of early pericytic biomarkers (Shi et al. 2020; French et al. 2025), have yet to be integrated into a unified framework that examines their specific relevance to sporadic AD and their translational potential.

This narrative review aims to address this gap by synthesizing updated evidence on BBB dysfunction in AD from human and animal studies published through March 2026 (Online Resource S1). We examine the reported temporal progression of BBB alterations, discuss proposed self-perpetuating feedback loops, and evaluate emerging vascular biomarkers and therapeutic approaches. This integration updates the state of the art and highlights the potential value of moving beyond a predominantly neurocentric, proteopathic perspective toward an integrated vascular strategy. Such an approach may offer a broader therapeutic window for the sporadic form of the disease, although further validation is required.

## Architecture and Functions of the BBB in the Healthy Brain

**Fig. 1:**
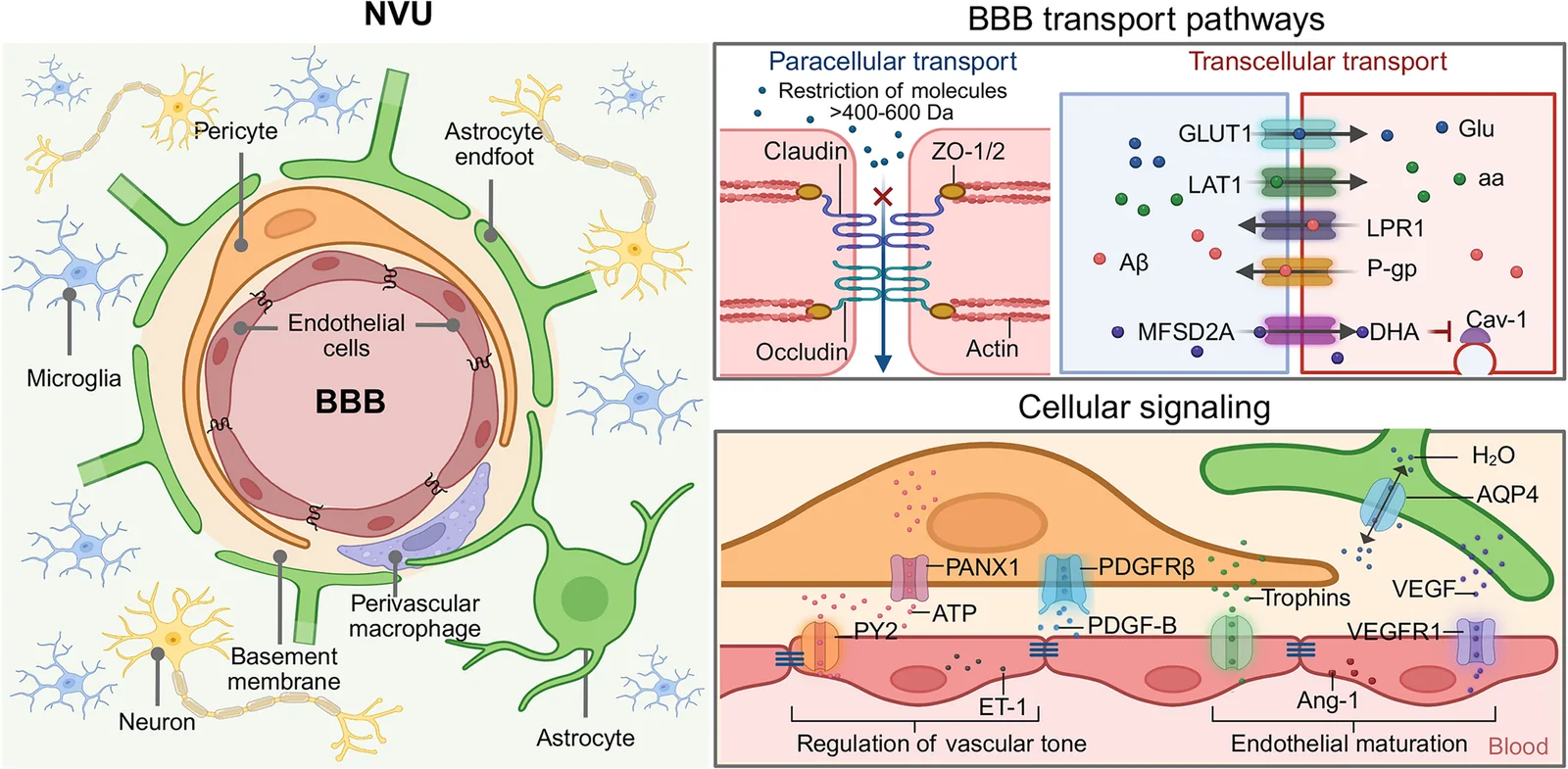
Schematic representation of the Neurovascular Unit (NVU) under physiological conditions (created with BioRender.com). Abbreviations: aa: amino acids; Ang-1: angiopoietin-1; AQP4: aquaporin-4; ATP: adenosine triphosphate; Aβ: amyloid-beta; BBB: blood-brain barrier; Cav-1: caveolin-1; DHA: docosahexaenoic acid; ET-1: endothelin-1; Glu: glucose; GLUT-1: glucose transporter 1; LAT1: L-type amino acid transporter 1; LRP1: low-density lipoprotein receptor-related protein 1; MFSD2A: major facilitator superfamily domain-containing protein 2 A; P2Y: P2Y receptors; PANX1: pannexin-1; PDGF-B: platelet-derived growth factor subunit B; PDGFRβ: PDGF-B receptor beta; P-gp: P-glycoprotein; VEGF: vascular endothelial growth factor; VEGFR-1: VEGF receptor 1; ZO-1/2: zonula occludens-1/2. Figure created in Biorender.com

The BBB, a highly specialized multicellular complex that regulates selective exchange between the systemic circulation and the brain parenchyma, is the core component of the neurovascular unit (NVU; Fig. 1). It comprises vascular endothelial cells, pericytes that stabilize capillaries, and astrocytes whose end-feet modulate blood flow and barrier properties. The NVU also integrates neurons, which regulate vascular tone through neurovascular coupling by releasing vasoactive signals in response to metabolic demand. Microglia and perivascular macrophages contribute to immune surveillance and vascular homeostasis, although they occupy distinct anatomical niches and perform partially overlapping functions (Kaplan et al. 2020; Schaeffer and Iadecola 2021; Mayer and Fischer 2024; Zhan et al. 2025). Under physiological conditions, this cellular network maintains ionic homeostasis and the central nervous system (CNS)’s energy supply while preventing the entry of macromolecules and pathogens (Stanimirovic and Friedman 2012; Soto-Rojas et al. 2021; Li et al. 2023; Gong et al. 2025).

This functional specialization results from the unique architecture of the cerebral endothelium. As the primary structural element of the BBB, these cells are characterized by the absence of fenestrations, a reduced number of pinocytotic vesicles, and, crucially, the formation of an intercellular tight junction complex (Daneman and Prat 2015; Xiang et al. 2025; Dotiwala et al. 2026). This molecular complex comprises transmembrane proteins, such as the dominant isoform claudin-5 (Cl5) and occludin, which are anchored to the cytoskeleton via cytoplasmic scaffolding proteins, such as zonula occludens-1 and − 2 (ZO-1/ZO-2). Tight junction integrity limits paracellular permeability to minimal values (below 0.0004 cm/s for small hydrophilic molecules; Fig. 1), establishing high transendothelial electrical resistance that necessitates solute transport through specific transcellular influx and efflux systems (Förster 2008; Luissint et al. 2012; Castro Dias et al. 2019).

Pericytes, recruited via platelet-derived growth factor subunit B (PDGF-B) and its receptor beta (PDGFRβ) signaling, envelop between 70% and 80% of the abluminal surface of the capillary endothelium (Bergers and Song 2005; Bell et al. 2010; Attwell et al. 2016; Uemura et al. 2020). In addition to providing structural stability, pericytes regulate the expression of Cl5 and ZO-1 via paracrine pathways and modulate vascular tone by releasing angiopoietin-1 (Shimizu et al. 2012). Beyond their structural role, pericytes actively control cerebral hemodynamics by regulating capillary diameter and perfusion in response to neuronal metabolic demand. Likewise, they act as trophic signaling hubs, releasing essential factors for endothelial survival and maturation, thereby consolidating the functional integrity of the NVU (Cheng et al. 2018; Hariharan et al. 2022).

Astrocytic endfeet, in turn, release soluble factors, including vascular endothelial growth factor (VEGF), sonic hedgehog, and retinoic acid, that induce and maintain the endothelial barrier phenotype (Guillamón-Vivancos et al. 2015; Manu et al. 2023; Padilla Gil 2024). Functionally, the BBB acts not only as a physical barrier but also as a polarized transport system. Influx transporters, such as glucose transporter 1 (GLUT-1) and L-type amino acid transporter 1 (LAT1), along with efflux transporters, such as P-glycoprotein and low-density lipoprotein receptor-related protein 1 (LRP1), ensure nutritional supply and active Aβ clearance (Zenaro et al. 2017; Wu et al. 2023). Caveolae-mediated transcytosis is another pathway that regulates BBB permeability. Caveolin-1 (Cav-1) is a structural component of caveolae, whereas major facilitator superfamily domain-containing protein 2 A (MFSD2A) suppresses caveolae formation and transcytosis, thereby supporting barrier integrity (Ben-Zvi et al. 2014). Concurrently, the glymphatic system and perivascular flow facilitate metabolite elimination into the cerebrospinal fluid (CSF) and meningeal lymphatic drainage (Chen et al. 2025b; Voumvourakis et al. 2026).

This multicellular and multifunctional architecture provides the brain with immunological and metabolic isolation. However, the same molecular complexity that ensures its efficacy also makes it a critical point of vulnerability. Subtle alterations in the expression or localization of any component can trigger a cascade of pathological events that, in the context of AD, can precede detectable protein accumulation and cognitive decline. This vulnerability appears particularly relevant in sporadic AD, where brain insulin resistance acts as an early contributing factor (Alkhalifa et al. 2023; Yin et al. 2026).

## Clinical Evidence in Humans: Temporal Pattern of BBB Dysfunction

**Fig. 2:**
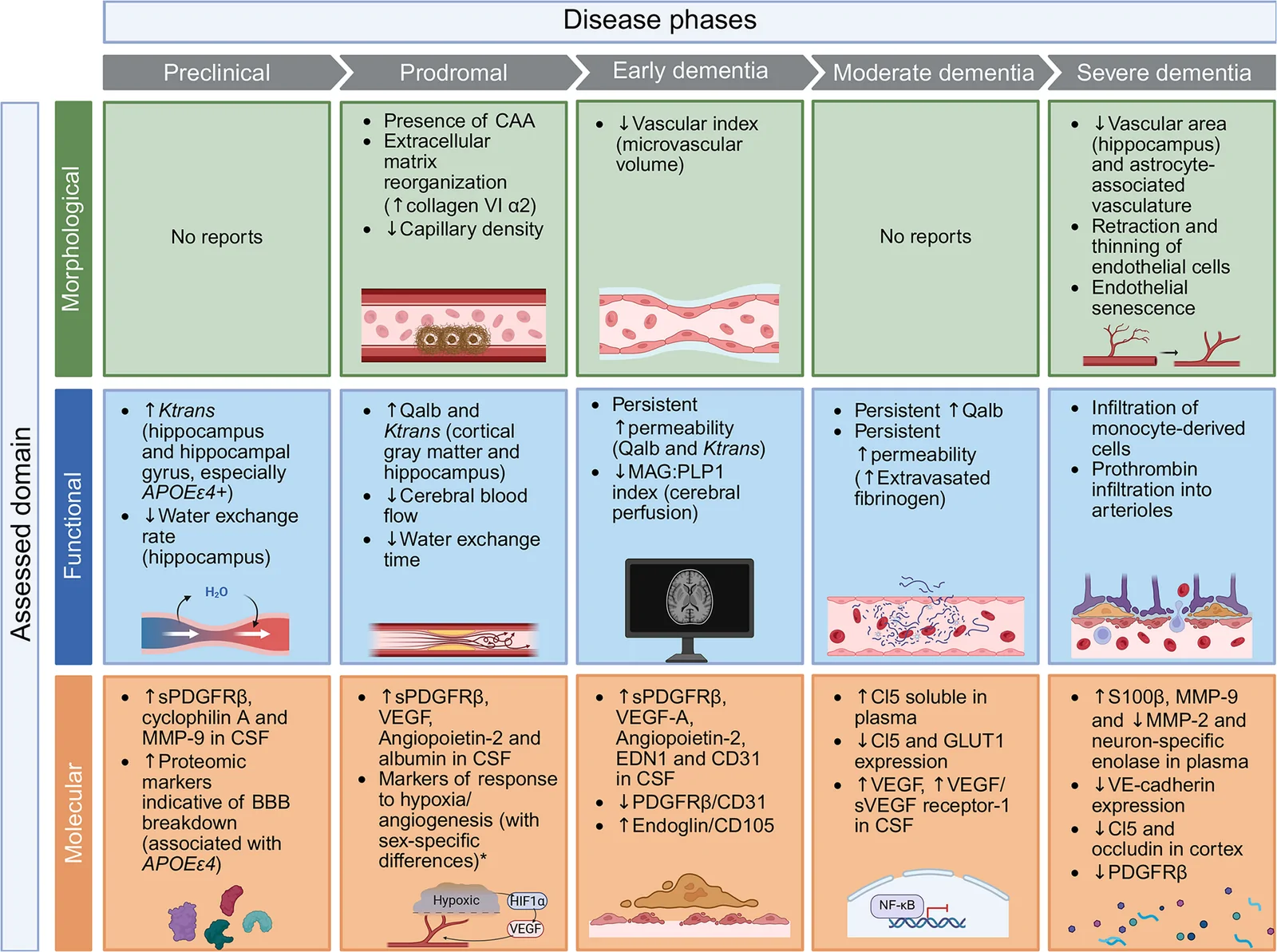
Temporal dynamics of morphological, functional, and molecular alterations of the blood-brain barrier (BBB) throughout the Alzheimer’s disease (AD) continuum, based exclusively on clinical evidence. Findings are organized into three domains: morphological (green), functional (blue), and molecular (orange; created with BioRender.com). Sex-specific findings are marked with an asterisk (*); the corresponding studies and endpoints are identified in Online Resource Table 1. Abbreviations: APOEε4: apolipoprotein E ε4; CAA: cerebral amyloid angiopathy; CD31: platelet endothelial cell adhesion molecule-1 (PECAM-1); CD105: endoglin; Cl5: claudin-5; EDN1: endothelin-1; GLUT1: glucose transporter 1; Ktrans: volume transfer constant (permeability rate); CSF: cerebrospinal fluid; MAG: PLP1: myelin-associated glycoprotein to proteolipid protein 1 ratio; MMP: matrix metalloproteinases; PDGFRβ: platelet-derived growth factor receptor beta; Qalb: CSF/plasma albumin ratio; sPDGFRβ: soluble PDGFRβ; S100β: S100 calcium-binding protein B; VE-cadherin: vascular endothelial cadherin; VEGF: vascular endothelial growth factor

BBB dysfunction is increasingly viewed, not simply as a late epiphenomenon of AD, but as an early and progressive process that appears to follow a relatively consistent chronology. Available evidence indicates that this process is most prominently detected in the hippocampus and may, in some cases, occur independently of substantial Aβ and tau burden during the initial stages (Montagne et al. 2015, 2020; Iturria-Medina et al. 2016; Tijms et al. 2021; Reas et al. 2024). Various studies using dynamic contrast-enhanced (DCE) magnetic resonance imaging (MRI) techniques, CSF and serum proteomics, and post-mortem analysis of cognitively healthy at-risk individuals *(APOEε4* carriers or biomarker-positive subjects), patients with MCI, and across different stages of AD dementia have helped outline this temporal sequence (Fig. 2, Online Resource Table 1). Findings are grouped into three domains (morphological, functional, and molecular) that appear to evolve stepwise, supporting the “two-hit” vascular hypothesis proposed for sporadic AD.

### Morphological Alterations

Unlike functional and molecular changes that can emerge during the preclinical phase, morphological alterations of the cerebral microvasculature have been documented primarily in symptomatic stages, mostly through descriptive or semi-quantitative approaches (Fig. 2, Online Resource Table 1). In cognitively healthy at-risk or preclinical individuals, few reports quantify ultrastructural modifications. These findings are limited to biomarkers indicative of vascular stress and subtle permeability increases detected by DCE-MRI in *APOEε4* carriers (Montagne et al. 2020; Visser et al. 2022; Wang et al. 2022a; Chen et al. 2025a; Vromen et al. 2025), with no clear evidence of pericyte degeneration, basement membrane thickening, or loss of astrocytic coverage.

The first relatively consistent morphological changes are described in the prodromal stage, characterized by thickening of the capillary basement membrane, vascular tortuosity, decreased vascular density, reduced capillary length between branching points, and signs of pericyte and endothelial cell degeneration, with several of these changes reported particularly in the hippocampus. These changes coexist with extracellular matrix reorganization (increased collagen VI α2) and cerebral amyloid angiopathy (CAA) (Wang et al. 2006; Baloyannis and Baloyannis 2012; Gallart-Palau et al. 2019; Leitner et al. 2024).

In more advanced stages, additional findings include reduced capillary density and vascular area (especially in the hippocampus); luminal narrowing; microvascular endothelial cell retraction and thinning; manifestations of endothelial senescence; infiltration of monocyte-derived cells into perivascular Aβ plaques; and a decrease in the vascular area associated with astrocytes (Zipser et al. 2007; Muñoz-Castro et al. 2023; Ting et al. 2023; Pomilio et al. 2024).

Importantly, the apparent regional predominance of early BBB alterations should not be interpreted as evidence that the BBB is spatially uniform elsewhere in the brain. Human endothelial cells exhibit region-specific molecular profiles, and studies of AD brains show regional differences in endothelial responses and BBB-related pathways across cortical areas (Bryant et al. 2023a). Thus, the hippocampus may represent one region of relatively early vascular vulnerability, but current evidence remains insufficient to establish a uniform spatial sequence of BBB dysfunction across the human brain.

Most of these findings derive from post-mortem studies employing descriptive or semi-quantitative techniques (immunohistochemistry, immunofluorescence, and electron microscopy), with limited application of rigorous morphometric analyses (normalized microvascular density, Feret diameter, tortuosity, capillary length, or astrocytic coverage). This limitation hinders the precise establishment of the temporal progression of each morphological change, its independent correlation with clinical severity, and reproducibility across cohorts. Nonetheless, the few studies incorporating quantitative analysis (Wang et al. 2006; Pomilio et al. 2024) suggest that microvascular loss in the hippocampus is a progressive feature that may precede or accompany cognitive decline.

The scarcity of quantitative morphological data in the preclinical phase, combined with the predominantly descriptive, cross-sectional nature of symptomatic-stage studies, represents a critical gap in the state of the art. This gap precludes discerning whether structural alterations constitute an early causal event or a secondary consequence of Aβ/tau pathology and limits the identification of precise therapeutic windows. Such limitations highlight the need for longitudinal, quantitative, and temporally controlled approaches.

### Functional Alterations

The most robust, quantitative evidence of BBB dysfunction comes from non-invasive or minimally invasive imaging techniques (Fig. 2, Online Resource Table 1). A subtle increase in BBB permeability is already detectable during the preclinical phase, evidenced by an elevation in the volume transfer constant (*Ktrans*, an indicator of contrast extravasation into the extravascular space) within the hippocampus and the hippocampal gyrus, particularly in *APOEε4* carriers, and by a decreased water exchange rate, measured via DCE-MRI and multi-echo arterial spin labeling (ASL) MRI, respectively (Montagne et al. 2020; Chen et al. 2025a).

These functional alterations appear to be amplified during the prodromal stage, where most studies report co-occurring increases in hippocampal and cortical gray matter *Ktrans* (more pronounced in *APOEε4* carriers), elevated water permeability, reduced cerebral blood flow, and an increased CSF/plasma albumin ratio (*Qalb*, a biochemical marker of BBB integrity) (Montagne et al. 2015; Lassila et al. 2018; Lin et al. 2021; Zhang et al. 2023; Reas et al. 2024; Sun et al. 2025; Padrela et al. 2026; Li et al. 2026). In early AD dementia, permeability increases selectively in the hippocampus (elevated *Ktrans* and *Qalb*), accompanied by signs of mild vasogenic edema (increased generalized free water detected by diffusion tensor imaging; DTI), cerebral hypoperfusion (decreased myelin-associated glycoprotein to proteolipid protein 1 (MAG: PLP1) ratio), and further evidence of BBB leakage, such as extravasated fibrinogen (Ji et al. 2017; Preis et al. 2024; Asby et al. 2025; Miners et al. 2025). In moderate and advanced stages, increased permeability persists (elevated *Qalb* and fibrinogen extravasation), chronic hypoperfusion worsens, and monocyte-derived cell infiltration and perivascular prothrombin accumulation become evident (Berzin et al. 2000; Zipser et al. 2007; Janelidze et al. 2017; Tayler et al. 2021; Wang et al. 2022b; Muñoz-Castro et al. 2023; Tian et al. 2024).

The regional pattern of these functional changes should be interpreted in the context of vascular-bed heterogeneity. Endothelial cells share a core BBB program across the CNS but also exhibit regional and arterial-capillary-venous specialization (Blanchette et al. 2025). Consequently, whole-brain or regional measurements of permeability and perfusion may combine alterations arising from distinct vascular compartments. Whether this heterogeneity contributes to the selective regional vulnerability observed during AD remains insufficiently established.

DCE-MRI has emerged as a leading method for the minimally invasive, quantitative assessment of regional permeability, while water-exchange MRI and DTI offer sensitive windows into early alterations in vascular homeostasis and vasogenic edema. Both parameters correlate with cognitive decline and, in several studies, appear to precede detectable Aβ/tau accumulation (Montagne et al. 2015, 2020; Ji et al. 2017; Zhang et al. 2023; Preis et al. 2024; Reas et al. 2024; Sun et al. 2025). These observations support the hypothesis that functional BBB breakdown may be an early event rather than solely a late consequence of parenchymal pathology.

Although neuroimaging has provided robust quantitative evidence of functional changes, most studies remain cross-sectional and lack longitudinal follow-up within the same subjects. This limitation, compounded by heterogeneity related to sex and *APOEε4* genotype, hinders the precise definition of therapeutic windows for intervention. Such gaps highlight the need for sporadic AD animal models that enable controlled, multidomain, and temporal characterization of BBB dysfunction.

### Molecular Alterations

Molecular biomarkers in serum/plasma represent the most accessible and least invasive evidence of BBB dysfunction (Fig. 2, Online Resource Table 1). During the preclinical phase, elevations are observed in proteins indicative of BBB breakdown (detected via untargeted proteomics), sPDGFRβ (a marker of pericytic damage), cyclophilin A, and matrix metalloproteinase-9 (MMP-9), the latter two being mediators of the enzymatic degradation of tight junctions and the basal lamina (Montagne et al. 2020; Visser et al. 2022; Wang et al. 2022a; Vromen et al. 2025).

During the prodromal stage, studies report increased CSF levels of sPDGFRβ, angiopoietin-2 (a BBB-destabilizing factor), albumin, and VEGF, along with decreased plasma-PDGFRβ in some reports; these findings correlate with AD pathology and are more evident in men. Furthermore, proteomics of isolated vessels reveals sexual dimorphism in angiogenesis- and hypoxia-related pathways, with an increase in hypoxia-response and angiogenesis markers in men, contrasted with a decrease in angiogenesis markers in women (Montagne et al. 2015; Sweeney et al. 2020; Lv et al. 2023; Van Hulle et al. 2024; Lin et al. 2024; Vrillon et al. 2025; Li et al. 2026). These findings suggest that sex differences may involve distinct vascular responses rather than a uniform difference in the magnitude of BBB dysfunction, with men showing greater activation of angiogenic and hypoxia-related pathways and women showing reduced expression of markers associated with microvascular repair.

In early-to-moderate AD dementia stages, key findings include increased levels of VEGF, placental growth factor, angiotensin-converting enzyme 1 (ACE-1), and fibrinogen, alongside a decreased PDGFRβ/CD31 ratio (indicative of pericyte-endothelium uncoupling). These changes are accompanied by reduced endothelial expression of Cl5 and GLUT1, with a concomitant increase in soluble Cl5 in plasma (Janelidze et al. 2017; Vogelsang et al. 2018; Wang et al. 2022b; Tachibana et al. 2024; Asby et al. 2025; Miners et al. 2025). In advanced stages, reports describe elevated plasma levels of LRP1, S100β, MMP-9, and perivascular prothrombin, as well as increased levels of inflammatory markers (IL-5, IL-17 A, IL-13, and IL-15). These findings coexist with reductions in vascular RAGE and MMP-2, and in the expression of VE-cadherin, Cl5, and occludin, alongside signs of endothelial senescence (Donahue et al. 2006; Yamazaki et al. 2019; Asby et al. 2021; Ting et al. 2023; Nehra et al. 2025). Such evidence suggests that molecular BBB alterations may evolve from an early, potentially maladaptive angiogenic phase toward more pronounced structural compromise, characterized by tight junction degradation, impaired toxin clearance, and chronic neuroinflammation.

To date, none of these biomarkers have been validated for clinical diagnosis or therapeutic monitoring. Nonetheless, fluid biomarkers (including CSF measures such as sPDGFRβ and angiopoietin-2, Qalb, and selected serum/plasma measures such as VEGF, MMP-9, MMP-2, and soluble Cl5) may support longitudinal assessment when interpreted according to the biological matrix. Their accessibility differs substantially because CSF-based measures require lumbar puncture, and their clinical utility requires independent validation.

Overall, the molecular, functional, and morphological findings are broadly consistent with a stepwise progression of BBB alterations throughout the disease. However, this progression may not be spatially uniform across brain regions or vascular compartments. Significant knowledge gaps persist. Morphological quantification remains scarce in most studies, and notable heterogeneity persists by sex and *APOEε4* genotype, with sex-dependent effects reported for several inflammatory and angiogenic markers. Furthermore, validated biomarkers that integrate morphological, functional, and molecular domains are still lacking. These gaps hinder translation into clinical practice and underscore the need for sporadic AD animal models that more faithfully reproduce the temporal sequence observed in humans.

## Evidence from Animal Models of AD: Reproducibility of BBB Dysfunction Chronology

**Fig. 3:**
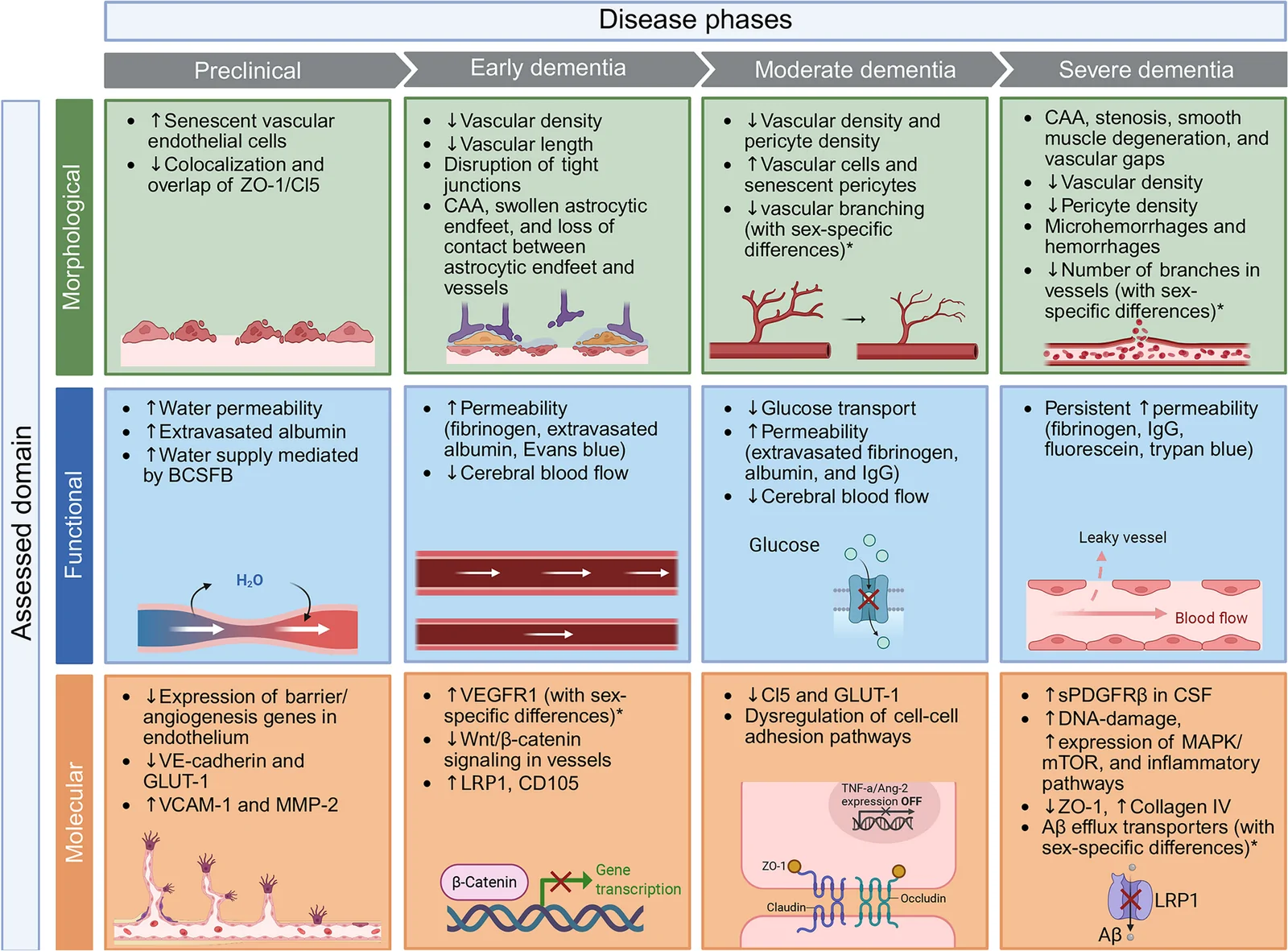
Temporal dynamics of morphological, functional, and molecular alterations of the blood-brain barrier (BBB) in animal models of Alzheimer’s disease (AD). Findings are organized into three domains: morphological (green), functional (blue), and molecular (orange; created with BioRender.com). Sex-specific findings are marked with an asterisk (*); the corresponding studies and endpoints are identified in Online Resource Table 2. Abbreviations: Aβ: amyloid-β peptide; BCSBF: blood-cerebrospinal fluid barrier; CAA: cerebral amyloid angiopathy; CD105: endoglin; Cl5: claudin-5; GLUT1: glucose transporter type 1; ICV-STZ: intracerebroventricular streptozotocin; IgG: immunoglobulin G; CSF: cerebrospinal fluid; LRP1: low-density lipoprotein receptor-related protein 1; MAPK: mitogen-activated protein kinase; MMP2: matrix metalloproteinase-2; mTOR: mammalian target of rapamycin; PDGFRβ: platelet-derived growth factor receptor beta; sPDGFRβ: soluble PDGFRβ; VCAM-1: vascular cell adhesion molecule-1; VE-cadherin: vascular endothelial cadherin; VEGF: vascular endothelial growth factor; ZO-1: zonula occludens-1

While transgenic models (primarily familial AD) have been extensively studied, evidence from inducible models of sporadic AD remains limited. Overall, BBB alterations exhibit variable reproducibility across models and time points. In several cases, these alterations parallel aspects of the progression reported in humans, particularly during the early stages (Fig. 3; Online Resource Table 2). The principal pathological features of the animal models included in this review are summarized in Online Resource Table 2.

### Morphological Alterations

In familial transgenic models (5XFAD, APP/PS1, 3xTg-AD, Tg2576, APP23, TgF344-AD, PDAPP-J20, PDAPP, APPSL, TgCRND8, P301S, p25, and ArcAβ), initial morphological changes have been reported as early as presymptomatic or very early phases (Fig. 3, Online Resource Table 2). These include: (i) an increase in senescent endothelial cells; (ii) loss of astrocytic end-foot contact with vessels; (iii) disruption of tight junctions; and (iv) reduced vascular density in the hippocampus and cortex (Merlini et al. 2011; Singh et al. 2021; Ting et al. 2023; Pomilio et al. 2024; Guzmán-Hernández and Fossati 2025). In moderate stages, findings include decreased pericyte density, vascular senescence, and reduced vascular branching in the neocortex, with some studies reporting greater reductions in females (Keyvani et al. 2018; Wu et al. 2019; Ting et al. 2023). Advanced stages are characterized by further reduction in vascular and pericytic density, severe CAA, microhemorrhages, vascular stenosis, smooth muscle degeneration in arteries or arterioles, and loss of astrocytic coverage (Merlini et al. 2011; Keyvani et al. 2018; Wu et al. 2019; Taccola et al. 2021; Bonaterra-Pastra et al. 2024; Pomilio et al. 2024; Lin et al. 2024; Karakatsani et al. 2025). In contrast, among the inducible models of sporadic AD identified within the scope of this review, intracerebroventricular streptozotocin (ICV-STZ) was the principal model for which BBB-related evidence was available, but its morphological characterization remains limited, with virtually no data regarding microvascular density, endothelial senescence, or astrocytic coverage in published cohorts (Gayger-Dias et al. 2024).

Regional differences in BBB vulnerability have also been examined in animal models, although these findings should be interpreted as complementary rather than directly equivalent to human evidence. In Tg2576 mice, in vivo sucrose uptake did not differ significantly between transgenic and wild-type animals in the hippocampus, cortex, or cerebellum, whereas uptake was increased in the olfactory bulbs. Global levels of Cl5, occludin, and ZO-1 were also similar between groups, although localized tight-junction alterations occurred near Aβ plaques (Nozohouri et al. 2025). These findings suggest that BBB dysfunction may be regionally heterogeneous and does not necessarily parallel the distribution of amyloid pathology.

The cerebellum is particularly informative in this context. Although it has traditionally been considered relatively resistant to classical AD proteinopathy compared with many cortical regions, structural alterations and gray matter loss have been reported across AD progression (Toniolo et al. 2018). Thus, cerebellar involvement should not be equated with either complete resistance to AD-related changes or with uniform BBB disruption. Instead, the available evidence suggests that regional neurodegeneration, protein deposition, and BBB dysfunction may follow partially distinct spatial trajectories.

### Functional Alterations

Increased BBB permeability is the most reproducible functional finding across models (Fig. 3, Online Resource Table 2). In the 5XFAD model, between 3 (presymptomatic) and 5 (early) months, an increase in permeability to water and Evans blue (a dye with high albumin affinity used to assess structural barrier integrity) has been reported, accompanied by reduced cerebral blood flow velocity (Zhang et al. 2024; Lin et al. 2024; Yao et al. 2026). In APP/PS1 and Tg2576 models, evidence of albumin, fibrinogen, and IgG extravasation into the brain parenchyma is present from very early stages (Singh et al. 2021; Wang et al. 2022b; Ting et al. 2023). Other models, such as TgF344-AD, APP23, 3xTg-AD, PDAPP-J20, ArcAβ, and PDAPP, exhibit hypoperfusion and increased permeability primarily in moderate to advanced stages (Merlini et al. 2011; Blockx et al. 2016; Bonaterra-Pastra et al. 2024; Pomilio et al. 2024; Soyer et al. 2024; Karakatsani et al. 2025). Conversely, the ICV-STZ model lacks quantitative functional assessments (DCE-MRI, cerebral perfusion, or tracer extravasation), a significant gap for evaluating its translational relevance.

### Molecular Alterations

In transgenic models, molecular analyses (Fig. 3, Online Resource Table 2) reveal early alterations in tight and adherens junction proteins (↓ZO-1, Cl5, occludin, and VE-cadherin), transporters (↓GLUT1, LRP1, and RAGE alterations), and signaling pathways (Wnt/β-catenin and immune response) (Löffler et al. 2016; Xiong et al. 2021; Singh et al. 2021; Wang et al. 2022b; Ting et al. 2023; Guzmán-Hernández and Fossati 2025; Yao et al. 2026). Models such as 5XFAD and TgCRND8 also show sex-dimorphic changes, with greater alterations in females reported for certain Aβ transport-related measures (Keyvani et al. 2018; Zhang et al. 2024).

The direction of sex effects differs across measures. In humans, some angiogenic and hypoxia-related responses appear more pronounced in men, whereas reduced expression of microvascular repair markers has been reported in women(Li et al. 2024, 2026). In animal models, studies report greater alterations in females for specific vascular morphological or Aβ transport-related endpoints (Keyvani et al. 2018). These findings do not establish a simple species-specific difference; rather, sex effects may depend on disease stage, biological process, vascular compartment, and experimental model.

Collectively, molecular and other BBB-related findings in familial AD transgenic models are broadly consistent with several aspects of the temporal progression of BBB dysfunction reported in humans, including early alterations in barrier integrity and transport systems, followed by more pronounced vascular and inflammatory changes at later stages. However, the timing and extent of these alterations vary across models, and their progression generally occurs over a much shorter timescale than in human AD. Moreover, these models primarily reflect the genetic mechanisms underlying familial AD.

In the ICV-STZ model, decreases in GLUT1 and claudin-2 (Cl2) have been reported during early stages, with decreased Cl5 reported at the moderate stage; however, integrated temporal evaluations addressing microvascular morphology, in vivo permeability, cerebral perfusion, and the expression of other tight junction proteins and pericytic markers across different phases of the model remain limited (Gayger-Dias et al. 2024; León-Arcia et al. 2026). This contrasts with the more extensive characterization available for transgenic models and leaves unresolved the extent to which the ICV-STZ model recapitulates the early vascular alterations observed in human sporadic AD.

Importantly, the ICV-STZ model remains one of the most widely used inducible approaches to study sporadic AD-like pathology. In parallel, the MODEL-AD consortium is developing models that incorporate human genetic risk factors associated with late-onset AD (Kotredes et al. 2021, 2024). Detailed evaluation of BBB alterations in these emerging models was beyond the scope of this review; however, their development expands the experimental framework for investigating vascular phenotypes relevant to late-onset AD.

## Molecular and Cellular Mechanisms Perpetuating BBB Breakdown

**Fig. 4:**
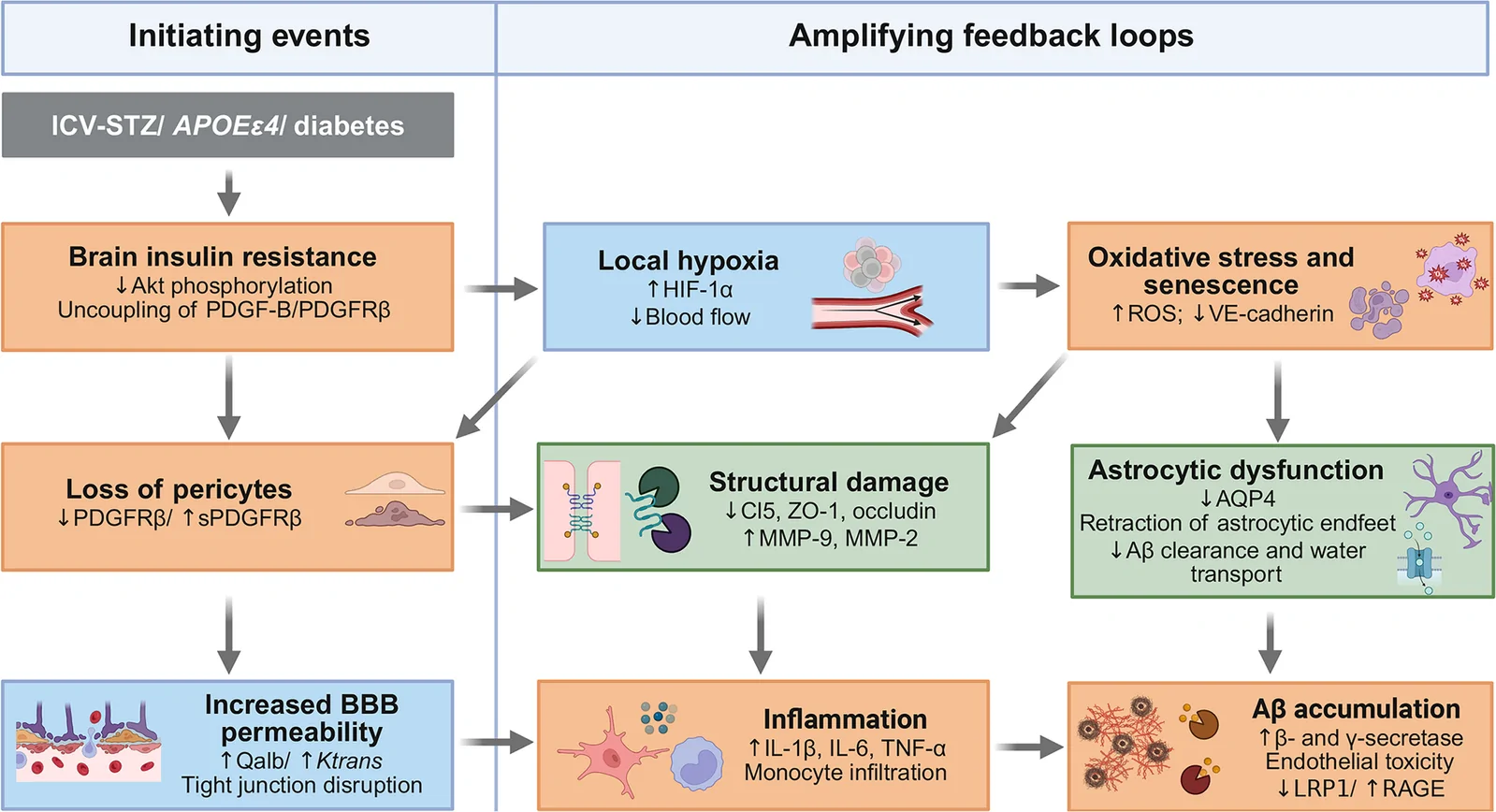
Simplified scheme of the main molecular and cellular mechanisms perpetuating blood-brain barrier (BBB) dysfunction in Alzheimer’s disease (AD). The left panel presents initiating events, including brain insulin resistance, PDGF-B/PDGFRβ uncoupling, pericyte loss, and increased BBB permeability. The right panel presents subsequent amplifying feedback loops involving structural damage, inflammation, hypoxia, oxidative stress, senescence, astrocytic dysfunction, and Aβ accumulation. Arrows indicate proposed, rather than definitively established, sequences and interactions (created with BioRender.com). Abbreviations: Aβ: amyloid-beta peptide; AQP4: aquaporin-4; APOEε4: apolipoprotein E ε4; Cl5: claudin-5; HIF-1α: hypoxia-inducible factor-1α; ICV-STZ: intracerebroventricular streptozotocin; IL: interleukin; Ktrans: volume transfer constant; LRP1: low-density lipoprotein receptor-related protein 1; MMP: matrix metalloproteinase; PDGFRβ: platelet-derived growth factor receptor beta; Qalb: CSF/plasma albumin ratio; RAGE: receptor for advanced glycation end-products; ROS: reactive oxygen species; sPDGFRβ: soluble PDGFRβ; TNF-α: tumor necrosis factor alpha; VE-cadherin: vascular endothelial cadherin; ZO-1: zonula occludens-1

BBB dysfunction in AD is neither isolated nor transient; available evidence suggests it can participate in an amplifying process maintained by interconnected molecular and cellular mechanisms. Once early breakdown is initiated, often associated with pericytic damage (Montagne et al. 2020; Wang et al. 2022a), these processes may form feedback loops that sustain permeability, hypoperfusion, and perivascular inflammation and may thereby contribute to neurodegeneration (Fig. 4). This framework is compatible with clinical and animal evidence summarized in Online Resource Tables 1 and 2, although the direction and temporal ordering of individual mechanisms remain incompletely established.

Pericyte loss has been reported during early stages of BBB dysfunction and has been linked to impaired PDGF-B/PDGFRβ signaling (Smyth et al. 2022; Guanqiao et al. 2023). Although the mechanisms remain incompletely defined, brain insulin resistance may contribute to this process in sporadic AD (Kellar and Craft 2020). Insulin signaling through PI3K/Akt helps maintain PDGFRβ expression at the pericyte membrane and limits apoptosis (Takahashi et al. 2015). When this signaling is disrupted, as occurs in the ICV-STZ model (Crunfli et al. 2018), Akt phosphorylation is reduced, promoting PDGFRβ internalization and degradation (Zhang et al. 2007; Er et al. 2013), as well as the release of its soluble form (sPDGFRβ) into the extracellular space. Insulin resistance also increases the production of reactive oxygen species (ROS) and activates MMP-9 and MMP-2, which can degrade the extracellular matrix and facilitate pericytic detachment (Underly et al. 2017; Yoon et al. 2023; Kim et al. 2024).

This initial pericyte loss compromises BBB integrity, increasing permeability (elevated *Ktrans* and Qalb, together with tight junction disruption). The resulting BBB breakdown may facilitate the entry of circulating inflammatory mediators (IL-1β, IL-6, TNF-α) and the infiltration of peripheral monocytes into the brain parenchyma. Once established, these events can trigger amplifying feedback loops involving local hypoxia, oxidative stress, endothelial senescence, and further vascular damage, thereby sustaining permeability and perivascular inflammation (Underly et al. 2017; Hayashi et al. 2020). Thus, brain insulin resistance may not only contribute to pericyte loss but also promote ongoing vascular dysfunction in sporadic AD (Fig. 4).

A second amplification pathway involves NVU dysregulation and astrocytic dysfunction. Retraction of astrocytic end-feet and loss of endothelial contact (documented in PDAPP-J20 and ArcAβ models from early stages; Online Resource Table 2) may compromise aquaporin-4 (AQP4)-dependent water transport and Aβ clearance (Peng et al. 2016; Silva et al. 2021). Concurrently, activation of perivascular microglia can release proinflammatory cytokines and ROS, promoting mitochondrial oxidative stress and endothelial senescence (Zhu et al. 2024; Li et al. 2025). This phenotype has been associated with reduced GLUT1 expression and may further exacerbate chronic hypoperfusion (Winkler et al. 2015).

Hypoxic signaling and pathological angiogenesis provide a third axis of amplification. Reduced cerebral blood flow and elevated hypoxia-inducible factor-1 alpha (HIF-1α) induce the transcription of VEGF and angiopoietin-2, which, while attempting to compensate for ischemia, can generate immature and leaky vessels (Qin et al. 2013; Zimna and Kurpisz 2015). This process may be further influenced by perivascular Aβ accumulation, which can affect endothelial and pericytic function and, under hypoxic conditions, may promote amyloidogenic processing through β- and γ-secretase activation (Alcendor 2020; Chen et al. 2024).

A key mechanism that may amplify this cycle is the imbalance in endothelial Aβ transporters characterized by decreased LRP1 (responsible for brain-to-blood efflux) and a simultaneous overexpression of RAGE (favoring blood-to-brain influx) (Donahue et al. 2006; Löffler et al. 2016). This pattern, reported in human post-mortem tissue and AD animal models (Online Resource Tables 1 and 2), is compatible with a direct positive feedback loop: reduced LRP1 limits vascular Aβ clearance and increases perivascular (Deng et al. 2026); while oligomeric Aβ can promote oxidative degradation of LRP1 (Owen et al. 2010) and stimulate RAGE expression through the nuclear factor kappa B (NF-κB) signaling (Chaney et al. 2005; Askarova et al. 2011). Brain insulin resistance may further influence this imbalance because PI3K/Akt signaling positively regulates LRP1 expression and membrane localization (He et al. 2021). However, studies that measure Aβ transporters together with structural and functional BBB changes remain limited; therefore, a self-sustaining transporter-mediated loop remains a mechanistic hypothesis rather than an established temporal sequence.

Finally, cellular senescence may contribute to the chronicity of the dysfunction (Knopp et al. 2023; Real et al. 2024). Senescent endothelial cells reported in APP/PS1 models and human post-mortem tissue (Ting et al. 2023) may reduce vascular repair capacity and sustain a proinflammatory state (Jia et al. 2019; Ramírez et al. 2022). Genetic and metabolic risk factors may further modify these mechanisms. In particular, *APOEε4* has been associated with BBB vulnerability and pericyte dysfunction through activation of the cyclophilin A/NF-κB/MMP-9 pathway, as well as with altered endothelial Aβ transport and clearance (Bell et al. 2012; Montagne et al. 2020; Kirchner et al. 2023). The magnitude and disease-stage dependence of these effects remain incompletely established. Hypertension, diabetes, brain insulin resistance, and glucotoxicity may interact with these pathways, although the ICV-STZ paradigm models only selected components of this complex risk architecture (Akinyemi et al. 2013; Scheffer et al. 2021; León-Arcia et al. 2026).

Beyond these candidate molecular pathways, single-cell and single-nucleus transcriptomic studies provide a broader view of vascular programs associated with AD. Analyses of human and experimental tissue have identified cell-type- and region-specific changes in endothelial cells, pericytes, and other NVU populations, including dysregulated angiogenic, inflammatory, metabolic, and barrier-related programs (Lau et al. 2020; Sun et al. 2023; Bryant et al. 2023b; Tsartsalis et al. 2024; Olayinka et al. 2025). These datasets can generate candidate molecular signatures for blood-based biomarker development and identify pathways amenable to therapeutic modulation. However, transcript abundance in brain vascular cells does not itself establish a circulating biomarker or actionable target; translation will require confirmation at the protein level, demonstration of vascular specificity, functional validation, and longitudinal association with disease progression.

These molecular and cellular mechanisms provide a framework for understanding how BBB breakdown may become self-sustaining. Identifying the points of convergence within these cycles, particularly early pericytic damage and dysregulated VEGF/angiopoietin signaling, may help define intervention points to interrupt pathological feedback before substantial neurovascular damage accumulates.

## Therapeutic Implications and Emerging Biomarkers: Toward a Vascular Strategy

Accumulating evidence from both human and animal models suggests that BBB dysfunction may serve as both an early biomarker and a potential therapeutic target in sporadic AD. Because vascular and BBB alterations may emerge before or evolve in parallel with some classical AD pathological changes, strategies aimed at preserving or restoring BBB integrity could, in principle, provide a therapeutic window extending into the preclinical stages of disease. By targeting the neurovascular feedback loops that may contribute to neurodegeneration, such approaches could complement established Aβ- and tau-directed strategies and potentially offer preventive or disease-modifying benefits, although clinical validation of BBB-directed interventions remains limited.

Among emerging biomarkers, those that reflect pericyte damage and early BBB permeability are of particular interest. CSF-sPDGFRβ is among the most studied indicators of pericyte injury and has been associated with increased hippocampal *Ktrans* and cognitive impairment, including in *APOEε4* carriers (Fig. 2, Online Resource Table 1). Importantly, these CSF findings should not be conflated with plasma PDGFRβ measurements, for which directionality and interpretation may differ across studies. Other candidates include angiopoietin-2, the CSF/plasma albumin ratio (Qalb), and serum MMP-9 and MMP-2 (Montagne et al. 2015, 2020; Van Hulle et al. 2024; Miners et al. 2025). These fluid and imaging measures may help identify patients with a larger vascular component, but none is currently validated as a diagnostic, surrogate, or pharmacodynamic endpoint for BBB-directed trials.

From a therapeutic perspective, vascular strategies have explored three main axes: (i) pericytic protection, (ii) restoration of insulin signaling, and (iii) modulation of pathological angiogenesis (Talbot 2014; Cheng et al. 2018; Nikolakopoulou et al. 2019; Georgieva et al. 2023; Zhang et al. 2024; Kieliszek-Ryba et al. 2026). In ICV-STZ and 5XFAD models, intranasal insulin or peroxisome proliferator-activated receptor gamma (PPARγ) agonists have been shown to restore Akt phosphorylation and improve vascular integrity (Rajasekar et al. 2017; Oh et al. 2022; Chen et al. 2023a; Zhang et al. 2024; Gendron et al. 2024). Selective MMP-9 inhibitors or tight-junction stabilizing approaches, including modulation of Wnt/β-catenin signaling, represent another avenue under investigation, particularly when combined with anti-inflammatory strategies targeting perivascular microglia (Aghaizu et al. 2020; Ringland et al. 2021; Wang et al. 2022b; Krawczuk and Mroczko 2026). Finally, selective blockade of VEGF or angiopoietin-2 has been proposed as a means of limiting the formation of immature, leaky vessels, preserving physiological angiogenesis, although the therapeutic window and safety of such approaches remain to be established (Coutelle et al. 2015; Lee et al. 2025; Liu et al. 2025; Kieliszek-Ryba et al. 2026). These approaches could potentially complement anti-Aβ therapies, particularly in patients with evidence of substantial vascular dysfunction, although their efficacy and optimal timing remain to be determined.

The relevance of vascular status is also underscored by the safety profile of clinically used anti-Aβ monoclonal antibodies. These agents can be associated with amyloid-related imaging abnormalities, including vasogenic edema (ARIA-E) and cerebral microhemorrhages or superficial siderosis (ARIA-H); cerebral amyloid angiopathy and *APOEε4* can increase this risk (Hampel et al. 2023; Alkhalifa et al. 2025). Pre-existing BBB and cerebrovascular alterations could plausibly influence antibody distribution, vascular exposure, clearance, efficacy, or safety. Nevertheless, BBB leakage does not necessarily produce proportionally greater parenchymal penetration of large biologics, and its specific effect on anti-Aβ antibody delivery remains incompletely established (Banks 2012; Sweeney et al. 2018; Han and Jiang 2021). These considerations support a complementary framework in which vascular status informs the selection and monitoring of anti-Aβ therapy rather than implying that BBB-directed and amyloid-directed strategies are competing approaches.

In this context, the ICV-STZ model remains of translational interest as a non-genetic approach that reproduces brain insulin resistance and several AD-like molecular and pathological alterations (Gayger-Dias et al. 2024; León-Arcia et al. 2026). Its potential value for investigating vascular mechanisms and interventions, however, remains to be established, given the limited characterization of BBB morphology and function across disease stages. In particular, quantitative assessments of microvascular architecture, in vivo BBB permeability, cerebral perfusion, and longitudinal pericytic alterations remain limited or unavailable. A comprehensive morphological, functional, and molecular characterization across preclinical, early, and advanced phases would help determine the extent to which this model can reproduce the vascular component of sporadic AD and provide a stronger basis for evaluating BBB-targeted interventions, including their potential to complement existing disease-modifying therapies.

## Limitations

Several limitations should be acknowledged. First, the most human studies reviewed are cross-sectional, which limits the strength of causal inferences about the temporal sequence of BBB changes. Longitudinal data that track the same individuals across preclinical, prodromal, and dementia stages remain scarce. Second, substantial methodological heterogeneity exists across studies, including differences in imaging protocols, biomarker assays, diagnostic criteria, and the degree of control for vascular comorbidities, sex, and *APOEε4* status. Third, quantitative morphometric analyses of the microvasculature in the preclinical phase are limited, and most morphological data derive from post-mortem descriptive or semi-quantitative approaches. Fourth, although familial AD transgenic models are extensively characterized, inducible models of sporadic AD (particularly ICV-STZ) still lack integrated morphological, functional, and molecular assessments across disease stages, limiting their translational validation as models of BBB dysfunction. Finally, as a narrative review, this work used non-systematic study selection and does not include a formal risk-of-bias assessment. These limitations underscore the need for carefully designed longitudinal human studies and more comprehensive characterization of sporadic AD models.

## Conclusions

The evidence analyzed suggests that BBB dysfunction is not merely a late event but a progressive process, with alterations in signaling, barrier integrity, and solute transport reported as early as the preclinical stage**s**. Available data indicate that this dysfunction may evolve toward structural impairment of the NVU, potentially contributing to chronic neuroinflammation and impaired Aβ clearance. Although aspects of this chronology have been reported in humans and transgenic models, the capacity of currently available sporadic AD models, particularly ICV-STZ, to fully reproduce the temporal and functional vascular component of the human disease remains incompletely established.

Underlying mechanisms, particularly the uncoupling of the PDGF-B/PDGFRβ pathway, which may be influenced by brain insulin resistance, may contribute to a self-reinforcing process of BBB dysfunction. Identifying feedback loops involving hypoxia, oxidative stress, and pathological angiogenesis positions NVU components as potential therapeutic targets that warrant further investigation.

Three questions should now be prioritized. First, does early BBB dysfunction contribute causally to the initiation and progression of neurodegeneration in sporadic AD, or does it primarily emerge downstream of amyloid and tau pathology? Second, which biological factors, including sex, *APOE*ε*4* genotype, metabolic status, and disease stage, are associated with distinct vascular trajectories across individuals? Third, which combination of fluid and imaging biomarkers can reliably identify distinct vascular trajectories before substantial and potentially irreversible neurovascular damage occurs?

Over the next five years, the most informative advances are likely to come from longitudinal multimodal studies combining advanced vascular imaging with plasma and CSF biomarkers, which may establish stage-specific vascular signatures and clarify the temporal relationship between BBB dysfunction and neurodegeneration. In parallel, systematic characterization of improved late-onset AD models may help determine which models most closely reproduce selected human vascular phenotypes and provide a stronger preclinical basis for testing BBB-targeted interventions. Finally, early clinical studies of vasculoprotective strategies, particularly in combination with established anti-amyloid approaches, could begin to clarify whether modifying vascular dysfunction influences disease progression.

Together, these advances could strengthen the evidence for BBB dysfunction as a measurable and potentially actionable component of AD pathophysiology, although substantial validation will still be required before vascular biomarkers or interventions can be incorporated into clinical practice.

## Supplementary Information

Below is the link to the electronic supplementary material.

Supplementary Material 1 (PDF 299 KB)

## Data Availability

All data analyzed and synthesized during this study are included in the manuscript and its supplementary information files.
