# Supplementary material for: “Morphological, Functional, and Molecular Progression of Blood-Brain Barrier Dysfunction in Alzheimer’s Disease”
Source: J Mol Neurosci. 2026 Oct 14;76(4):153. doi: 10.1007/s12031-026-02610-0 (PMC13669046; doi:10.1007/s12031-026-02610-0)
Supplement: Supplementary file 1 [file 12031_2026_2610_MOESM1_ESM.pdf]

Supplementary materials

Title: "Morphological, functional, and molecular progression of blood-brain barrier breakdown in Alzheimer's disease"

Authors: Karen León-Arcia[abc], Humberto Martínez-Orozco[a], Magdalena Guerra-Crespo[d], Sofía Y. Díaz-Miranda[a]*, Luis O. Soto-Rojas[b]*

Affiliation:

[a] Departamento de Neurobiología del Desarrollo y Neurofisiología, Instituto de Neurobiología, Universidad Nacional Autónoma de México (UNAM), Querétaro, CP 76230, México.

[b] Laboratorio de Investigación en Neurociencias y Enfermedades Neurodegenerativas (LINEN), Facultad de Estudios Superiores de Iztacala, UNAM, CP 54090, México.

[c] PhD student, Posgrado en Ciencias Biológicas, Unidad de Posgrado, Edificio A, 1er Piso, Circuito de Posgrado, Ciudad Universitaria, UNAM, Ciudad de México, CP 04510, México.

[d] Laboratorio de Medicina Regenerativa, Departamento de Fisiología, Facultad de Medicina, UNAM, Ciudad de México, CP 04510, México.

***Corresponding authors:** Sofía Y. Díaz-Miranda and Luis O. Soto-Rojas

S1. Literature search strategy

A systematic search was conducted in the PubMed database on April 2, 2026, without publication date restrictions or additional filters. The following search equation was utilized:

("blood-brain barrier" OR BBB OR "barrera hematoencefálica") AND (Alzheimer OR "Alzheimer's disease") AND (dysfunction OR permeability OR breakdown OR leakage OR "tight junction") AND (human OR patient OR postmortem OR CSF OR imaging OR DCE-MRI) AND (MCI OR "mild cognitive impairment" OR preclinical OR stage OR chronology OR timeline)

The search yielded 542 documents, of which 255 corresponded to systematic reviews, meta-analyses, or literature reviews.

Inclusion Criteria:

- Original research articles describing evidence of blood-brain barrier (BBB) dysfunction in patients with Alzheimer's disease or its prodromal stages, or in animal models of the disease.
- Studies that include a healthy control group and explicitly compare patients versus controls.

Exclusion Criteria:

- Retracted articles.
- Comments, reviews, meta-analyses, and non-peer-reviewed articles.
- Exclusively *in vitro* studies.
- Studies without a healthy control group.
- Studies providing no information on the clinical stage of the disease (preclinical, MCI, early, moderate, or advanced AD).

The selected articles (n = 68) were used to develop Table 1 (human findings) and Table 2 (animal model findings).

Table 1. Temporal progression of blood-brain barrier alterations during Alzheimer's disease progression. *Note: Clinical stage categorization is based on the National Institute on Aging and Alzheimer's Association (NIA-AA) diagnostic criteria.

Disease stage *	Morphological alterations	Functional alterations	Molecular alterations/ biomarkers	Main technique	DOI
Preclinical (cognitively normal at risk)			↑Proteins indicative of BBB disruption (associated with *APOEε4*) and alterations in glucose metabolism	Non-targeted proteomics (tandem mass labeling mass spectrometry) in CSF	10.1002/alz.70738
Preclinical (cognitively normal at risk)		↓Water exchange rate in the hippocampus		MRI	10.1002/alz.70089
Preclinical (cognitively normal at risk)			↑Proteins indicative of BBB and blood-CSF barrier dysfunction	Proteomics and genomics	10.1186/s13024-022-00521-3
Preclinical (cognitively normal at risk)			↑CSF-sPDGFRβ	ELISA	10.1186/s13024-021-00512-w
Preclinical (cognitively normal at risk)		↑*Ktrans* (↑permeability) in the hippocampus and hippocampal gyrus of *APOEε4+*	↑CSF-sPDGFRβ, ↑CSF-ciclofilin A, ↑CSF-MMP-9	DCE-MRI Immunoassays	10.1038/s41586-020-2247-3
Prodromal (MCI)		↑Qalb (↑permeability)	↓Plasma-PDGFRβ (correlated with AD pathology, stronger in men)	ELISA	10.1186/s13024-026-00926-4
Prodromal (MCI)		↓Water exchange time		Multi-echo arterial spin marking (MRI)	10.1016/j.nicl.2025.103926

Prodromal (MCI)		↓Cerebral blood flow, ↑*Ktrans* (↑permeability)		DCE-MRI	10.5498/wjp.v15.i6.103661
Prodromal (MCI)		No changes in Qalb	↑CSF-PDGFRβ	ELISA	10.1016/j.ebiom.2025.105694
Prodromal (MCI)		↑Ktrans (↑permeability) in cortical gray matter, especially in *APOEε4* carriers		DCE-MRI	10.1002/alz.14302
Prodromal (MCI)	↑Collagen VI α2 in vessels (reorganization of the extracellular matrix), presence of amyloid angiopathy		↑SEMA3G (specific marker of amyloid angiopathy)	Laser capture microdissection of vessels and mass spectrometry	10.1007/s00401-024-02767-1
Prodromal (MCI)			↑Markers of response to hypoxia and angiogenesis in men; ↑antigen presentation and ↓angiogenesis in women	Isolated vessel proteomics	10.1111/acel.14264
Prodromal (MCI)			↑CSF-Angiopoietin-2, CSF-albumin	ELISA	10.1038/s41398-023-02706-w
Prodromal (MCI)			↑CSF-sPDGFRβ	Immunoassays	10.1186/s13195-023-01199-5, 10.1002/alz.12061
Prodromal (MCI)		↑Vascular water exchange in the hippocampus		MRI	10.1016/j.neuroimage.2023.119951
Prodromal (MCI)			No alteration in CSF-PDGFRβ	ELISA	10.1002/alz.12506

Prodromal (MCI)			↓serum-PTPRB, ↓serum-GJA5, ↑serum-KCNJ8, ↑serum-vWF, and correlate with amyloid deposition	ELISA	10.3233/JAD-215135
Prodromal (MCI)		↑Product of water permeability and surface area (↑water permeability), without modification of Qalb		MRI and turbidimetric assay	10.1002/ana.26134
Prodromal (MCI)	↑Expression of α-4-like angiopoietin concentrated in regions near vessels with amyloid angiopathy, ↑microvascular density in tissues with amyloid angiopathy		↑Expression of EGF-containing fibulin, ↑OGFRL1, ↑ubiquitin protein ligases (RNF114, RNF181 and UBE2), ↑vacuolar VPS37C proteins	Mass spectrometry	10.1111/bpa.12699
Prodromal (MCI)		↓Cerebral blood flow		Blood pressure monitoring using a Holter monitor and carotid ultrasound imaging	10.1109/TBME.2017.2759511
Prodromal (MCI)		No changes in Qalb	↑CSF-VEGF, ↑CSF-VEGF/sVEGFR-1	Immunoassay (multiplex electrochemiluminescence)	10.1016/j.neurobiolaging.2016.11.017
Prodromal (MCI)			↑serum-HMGB1,	ELISA	10.1186/s12974-016-0670-z
Prodromal (MCI)	↑Tortuosity, ↑capillary length between branching points, ↓capillary density/neuronal density, degeneration of pericytes and			Golgi staining and EM	10.1016/j.jns.2012.07.010

	endothelial cells, proliferation of perivascular microglia, presence of pinocytic vesicles and cytoplasmic inclusions in pericytes and endothelial cells, ↓length of tight junctions and number of tight junctions per vessel length, ↑thickness of the basement membrane, ↓capillary density, ↓diameter and number of mitochondria of endothelial cells, especially in the hippocampus				
Prodromal (MCI)		↑Ktrans (↑permeability) only in the hippocampus ↑Qalb (↑permeability)	↑CSF-sPDGFRβ, no alterations in sICAM-1, sVCAM-1, or MMP-9	DCE-MRI	10.1016/j.neuron.2014.12.032
Prodromal (MCI)	↓Vascular index (vascular volume)	No differences in the permeability index		DCE-MRI	10.1002/jmri.20669
Prodromal (MCI)		No alterations in Qalb			10.1002/alz.13433
AD dementia (early)		↑Generalized free water (mild vascular changes)		Diffusion tensor imaging (DTI)	10.1186/s13195-017-0292-4
AD dementia (early)		↑Qalb (↑permeability)	↑CSF-Angiopoietin-2, ↑CSF-placental growth factor, ↑CSF-ACE-1,	ELISA	10.1002/alz.70957
AD dementia (early)		↓MAG:PLP1 index (↓cerebral perfusion), ↑Fibrinogen (↑permeability)	↑VEGF-A, ↑EDN1, ↑CD31, ↓PDGFRβ/CD31, ↑endoglin/CD105 (↑proliferation of endothelial cells)	ELISA	10.1093/brain/awaf394

AD dementia (early)			↑proteins indicative of BBB dysfunction	Non-targeted proteomics (tandem mass-labeled mass spectrometry) in CSF	10.1093/brain/awaf251
AD dementia (early)		↑*Ktrans* (↑permeability)	No alterations in CSF-PDGFRβ	DCE-MRI<br>ELISA	10.1186/s13195-024-01529-1
AD dementia (early)			↓Glut1 expression in brain-derived endothelial cells in the blood	Flow cytometry	10.1016/j.brainres.2017.10.035
AD dementia (early)		No change in permeability		DCE-MRI	10.1016/j.pscychresns.2008.04.003
AD dementia (moderate)			↑plasma-Cl5	Ultrasensitive immunoassay (SIMOA)	10.3390/ijms25031419
AD dementia (moderate)		↑Extravasated fibrinogen (↑permeability)	↓Cl5 and GLUT1 expression	IHC	10.1093/brain/awac236
AD dementia (moderate)		↑Qalb (↑permeability)	↑CSF-VEGF, ↑CSF-VEGF/sVEGF receptor-1	Immunoassay (multiplex electrochemiluminescence)	10.1016/j.neurobiolaging.2016.11.017
AD dementia (moderate)			↑Soluble serum-receptor endothelial protein C	ELISA	10.1177/153331751 4567124
AD dementia (moderate-severe)		↑Qalb (↑permeability)		Automatic protein analyzer	10.1016/j.archger.2024.105441
AD dementia (moderate-severe)		Perivascular prothrombin	↑Agrin (basement membrane) in the hippocampus and the prefrontal cortex	ELISA	10.1016/s0197-4580(00)00121-4
AD dementia (severe)	No differences in microvasculature diameter, nor expression of Cl5, ZO-1, or VCAM-1		↑plasma-S100β, ↑plasma-MMP-9, ↓plasma-MMP-2, ↓plasma-neuron-specific enolase	ELISA<br>IF	10.1186/s12987-024-00615-8

AD dementia (severe)	↓Vascular area in the hippocampus, ↓area of the vasculature associated with astrocytes			IF, IHC	10.1007/s12035-023-03897-5
AD dementia (severe)	Presence of senescent endothelial cells		↓VE-cadherin expression	IF	10.1007/s11357-023-00927-x
AD dementia (severe)	Presence of monocyte-derived cells in hippocampal Aβ plaques in the vicinity of blood vessels	Infiltration of monocyte-derived cells	↑Expression of genes related to endothelial activation	IHC IF RT-qPCR	10.1186/s40478-023-01530-z
AD dementia (severe)			↑IL-5, ↑IL-17A, ↑IL-13, ↑IL-15, ↓MAG:PLP1, ↓PDGFRβ, ↑fibrinogen, ↑EDN1 in the brain tissue	ELISA	10.1093/brain/awab094
AD dementia (severe)		↓MAG:PLP1 in parietal and frontal white matter, ↑fibrinogen in parietal and frontal cortex		ELISA	10.1111/bpa.12935
AD dementia (severe)			↓Cl5, ↓occludin in the cortex	ELISA	10.1093/brain/awz011
AD dementia (severe)	Retraction and thinning of microvascular endothelial cells	Prothrombin infiltration into arterioles	↑Prothrombin expression in the cortex	IHC IF ELISA	10.1016/j.neurobiolaging.2006.05.016
AD dementia (severe)	Colocalization of LPR1 and Aβ		↑RAGE expression, ↓LPR1 in the hippocampus	IF	10.1007/s00401-006-0115-3

Abbreviations: *ACE-1: angiotensin-converting enzyme-1, APOE: apolipoprotein E, Aβ: β-amyloid, CD105: endoglin, CD31: endothelial cell and platelet adhesion molecule-1, Cl 5: Claudin 5, DCE-MRI: dynamic contrast magnetic resonance imaging, MCI: mild cognitive impairment, AD: Alzheimer's disease, EDN1: endothelin-1, ELISA: enzyme-linked immunosorbent assay, GJA5: gap junction alpha-5 protein, GLUT: glucose transporter, HMGB1: high mobility cluster protein 1, IF: immunofluorescence, IHC: immunohistochemistry, IL: interleukin, KCNJ8. adenosine triphosphate-8 sensitive internal rectifying potassium channel, Ktrans: contrast transfer rate, CSF: cerebrospinal fluid, MAG:PLP1: myelin-associated glycoprotein:proteolipid protein-1 ratio, MMP: metalloproteinases, OGFRL1: opioid growth factor receptor, PDGFRβ: platelet-derived growth factor receptor beta, PTPRB: protein tyrosine phosphatase type B receptor, Qalb: CSF/Plasma albumin ratio, sRAGE: soluble*

receptor for advanced glycation end products, MRI: nuclear magnetic resonance, RT-qPCR: quantitative reverse transcription polymerase chain reaction, SEMA3G: semaphorin 3G, SIMOA: single molecule array, sPDGFRβ: (soluble PDGFRβ), VCAM-1: vascular cell adhesion molecule-1, VE: vascular endothelium VEGF: vascular endothelial growth factor, vWF: von Willebrand factor.

Table 2. Reproducibility of changes in the BHE in animal models of Alzheimer's disease.

Animal model (model type)	AD pathology	Time point (stage)	Morphological alterations	Functional alterations	Molecular alterations	DOI
5XFAD (Transgenic /familial)	Aβ	3 months (presymptomatic)		↑Water molecule permeability; ↑permeability area; ↓oxygen extraction fraction	↓Endothelium barrier/angiogenesis genes; ↑astrocyte immune response genes; no changes in the expression of Cl5, Occludin, CD31 or AQP4	10.1186/s12987-025-00756-4
5XFAD (Transgenic /familial)	Aβ	4 months (early)	↓Vascular density in the retrosplenial cortex and hippocampus, ↓vascular length	↓Blood flow velocity, ↑permeability in the hippocampus		10.1016/j.ebiom.2024.105355
5XFAD (Transgenic /familial)	Aβ	5 months (early)	↑Number of neutrophils attached to the vessels	↑Permeability in cortex and hippocampus (Evans blue), ↓cerebral blood flow	↓Soluble VEGFR1 in females, ↑VEGFR1 (full chain) in males. Changes in genes associated with blood-brain barrier integrity and vascular smooth muscle	10.1186/s40035-023-00388-4
5XFAD (Transgenic /familial)	Aβ	6 months (moderate)		↑Extravasated fibrinogen (↑permeability)		10.3390/ijms23137320
5XFAD (Transgenic /familial)	Aβ	7 months (moderate) y 14 months (severe)	↓Vascular density in the retrosplenial cortex of the hippocampus and visual cortex	↓Blood flow velocity, ↑permeability in the hippocampus		10.1016/j.ebiom.2024.105355
5XFAD (Transgenic /familial)	Aβ	12 months (severe)			Differentially expressed genes in DNA damage pathways, immune reactivity, inflammation, MAPK, mTOR, endocytosis, and GWAS risk genes for human AD	10.1186/s13195-017-0292-4
5XFAD (Transgenic /familial)	Aβ	13 months (severe)		↑Fibrinogen, ↑permeability index (area of extravasated 4 or	↓ZO-1 expression in the cortex but not in the hippocampus	10.1016/j.ymthe.2025.08.024

				70 kDa dextran/area of vessels)		
3xTg-AD (Transgenic /familial)	Aβ + tau	2 months (presymptomatic)-8 months (moderate)		↑Water supply mediated by BCSBF		10.1186/s12987-024-00597-7
3xTg-AD (Transgenic /familial)	Aβ + tau	17.5 months (severe)	Presence of cerebral amyloid angiopathy	↑permeability (DCE-MRI)		10.1002/alz.14511
3xTg-AD (Transgenic /familial)	Aβ + tau	26 months (severe)		No changes in paracellular permeability (no alteration in [14C]Sucrose perfusion)		10.1186/s12987-019-0158-1
APP23 (Transgenic /familial)	Aβ	22 months (severe)	Presence of microhemorrhages and hemorrhages, ↓Smooth muscle actin in vessels	↑Fibrinogen in blood vessels (↑permeability)		10.1186/s13195-024-01541-5
APP/PS1 (Transgenic /familial)	Aβ	2 months (presymptomatic)	↑Density of senescent vascular endothelial cells	↑Albumin extravasated into small vessels (↑permeability)	↓VE-cadherin expression in vessels	10.1007/s11357-023-00927-x
APP/PS1 (Transgenic /familial)	Aβ	3 months (early)			↓Expression of ZO1 and Cl5	10.1002/ptr.6849
APP/PS1 (Transgenic /familial)	Aβ	4 months (early)		↑Extravasated fibrinogen (↑permeability)	↓Expression of Cl5 and GLUT1, ↓Wnt/β-catenin signaling in vessels	10.1093/brain/awac236
APP/PS1 (Transgenic /familial)	Aβ	6 months (moderate)	↓Pericyte density	↑Extravasated IgG		10.18632/aging.102171
APP/PS1 (Transgenic /familial)	Aβ	8 months (moderate)	↑Density of vascular cells and senescent pericytes	↑Extravasated albumin (↑permeability)	↓Cl5 expression in vessels, alteration in cell-cell adhesion pathways	10.1007/s11357-023-00927-x
APP/PS1 (Transgenic /familial)	Aβ	9 months (severe)	↓Pericyte density	↑Extravasated IgG		10.18632/aging.102171
TgF344-AD (Transgenic /familial)	Aβ + tau	9 months (moderate)		↑Permeability ([18F]FDG-PET)		10.1016/j.pharma.2024.04.004
PDAPP-J20 (Transgenic /familial)	Aβ	5 months (early)	↓Positive area to occludin			10.1007/s12035-023-03897-5
PDAPP-J20 (Transgenic /familial)	Aβ	15 months (severe)	↑Wrinkled and narrowed blood vessels,	↑Permeability (fluorescein perfusion)		

			↓%vessel-associated astrocytes			
PDAPP (Transgenic /familial)	Aβ	18 months (severe)		↑Permeability (GD-DOTA perfusion)		10.3233/JAD-160023
APP_{SL} (Transgenic /familial)	Aβ	6 months (early)			↑LPR1 expression (Aβ efflux), without changes in occludin, CD31, ZO-1, or VCAM1	10.3389/fnins.2016.00232
Tg2576 AD (Transgenic /familial)	Aβ	10 months (early)	↑% disruption of tight junctions	↑Extravasated albumin (↑permeability)	↑CD105 expression, ↓ZO1 and occludin expression	10.1016/j.ebiom.2021.103503
		16 months (severe)			↑CSF-sPDGFRβ	10.1016/j.neuron.2014.12.032
TgCRND8 (Transgenic /familial)	Aβ	6 months (moderate)	↓The number of branches in neocortical vessels, more pronounced in females		↓Expression of Aβ transporters (efflux): MDR1 and CLU, with no change in the expression of RAGE (influx)	10.1111/bpa.12599
TgCRND8 (Transgenic /familial)	Aβ	12 months (severe)	↓Number of branches in vessels of the neocortex, hippocampus and basal ganglia, more pronounced in females		↓Expression of Aβ transporters (efflux): CLU, no change in the expression of RAGE (influx)	
P301S (Transgenic /familial)	Tau	3 months (presymptomatic)	↓Colocalization and overlap of ZO-1/Cl5		↑Expression of VCAM-1, GLUT1, and MMP2	10.1002/alz.70077
p25 (Transgenic /familial)	Neurodegenerative	3 weeks (early)	Without alterations in vascular density and cerebrovascular volume	Without alterations in the passive diffusion of [3H]-diazepam or the extravasation of [14C]-sucrose, ↓amino acid transport ([3H]-phenylalanine)	No changes in the expression of Cl5, ZO-1, GLUT-1 and occludin	10.1016/j.neuropharm.2021.108588
p25 (Transgenic /familial)	Neurodegenerative	6 weeks (severe)	↑Vascular density (GLUT-1+ cells) ↓cerebrovascular volume	Without alterations in the passive diffusion of [3H]-diazepam or the extravasation of [14C]-sucrose, ↓glucose transport (↓[3H]-d-glucose cerebral)	↑Expression of collagen IV, altered expression of Cl5, ZO-1, and LAT-1	
ArcAβ (Transgenic /familial)	Aβ	6 months (early)	Vascular Aβ deposits, swollen astrocytic foot processes, and			10.1007/s00401-011-0834-y

			loss of contact between astrocytic foot processes and vessels. Other morphological parameters were unremarkable			
ArcAβ (Transgenic /familial)	Aβ	9-13 months (moderate)	Vessel morphology without evident alteration		↓GLUT1 expression, no change in CD31 expression	
ArcAβ (Transgenic /familial)	Aβ	16-22 months (severe)	Severe amyloid angiopathy, swollen astrocytic foot-and-socket lesions and loss of contact of the astrocytic foot-and-socket lesions with the vessels, stenosis and bark-like walls in arteries and arterioles, presence of gaps in the vascular bed, presence of microhemorrhages, constrictions/stenosis and thickening of the vessel walls with amyloid angiopathy, smooth muscle degeneration with amyloid lymphadenopathy	Extravasation of IgG, perfusion resin, and trypan blue	↓Expression of astrocytic β-dystroglycan	
ICV-STZ (Inducible)	Sporadic AD-like	2 weeks (early)			↓GLUT1 and Cl2 expression, but not Cl5 expression	10.1007/s11064-024-04144-6
ICV-STZ (Inducible)	Sporadic AD-like	4 weeks (moderate)			↓GLUT1, Cl2, Cl5 expression	

Abbreviations: *AQP4: aquaporin-4, BCSBF: blood-cerebrospinal fluid barrier, CD31: platelet endothelial cell adhesion molecule-1 (PECAM-1), Cl2: claudin-2, Cl5: claudin-5, CLU: clusterin, DCE-MRI: dynamic contrast-enhanced magnetic resonance imaging, GD-DOTA: gadoterate meglumine, GLUT1: glucose transporter type 1, IgG: immunoglobulin G, CSF: cerebrospinal fluid, LAT-1: large neutral amino acid transporter 1, MDR1: multidrug resistance protein 1, MMP2: matrix metalloproteinase 2, RAGE: receptor for advanced glycation end products, sPDGFRβ: soluble platelet-derived growth factor receptor beta, VCAM-1: vascular cell adhesion molecule-1, VE-cadherin: vascular endothelial cadherin, VEGFR1: vascular endothelial growth factor receptor 1 vascular, ZO-1: zonula occludens-1, [^{18}F]FDG-PET: ^{18}F-fluorodeoxyglucose positron emission tomography; radioactive markers used as tracers: [^{3}H]-diazepam, [^{14}C]-sucrose, [^{3}H]-phenylalanine, [^{3}H]-d-glucose*
